# Supplementary material for: High prevalence of cardiovascular risk factors in pregnant women in Benin
Source: PLoS One. 2024 Nov 14;19(11):e0310408. doi: 10.1371/journal.pone.0310408 (PMC11563467; doi:10.1371/journal.pone.0310408)
Supplement: S1 Table — (DOCX) [file pone.0310408.s001.docx]

**Table**: Factors associated with hyperglycemia and obesity in pregnant women

| **Hyperglycemia** | | | | | | |
| --- | --- | --- | --- | --- | --- | --- |
|  | **Univariate** | | | | **Mulivariate** | |
|  | **n(%)** | **OR [IC95%]** | | **P** | **OR [IC95%]** | **p** |
| **Age classes** | |  | | **0.2904** |  |  |
| 15 – 24 | 60(14.8%) | 1.3[0.9-2.0] | | 0.1480 | 1.3[0.9-2.0] | .1757 |
| 25 – 34 | 57(11.5%) | 1 | | - |  | .7422 |
| ≥ 35 | 12(11.0%) | 1.0[0.5-1.8] | | 0.8810 | 0.9[0.4-1.7] |  |
| **Area** | |  | | **0.0118** |  |  |
| Urban | 73(10.8%) | 1 | | - |  | - |
| Rural | 56(16.7%) | 1.6[1.1-2.4] | | 0.0093 | 1.4[1.0-2.1] | .0758 |
| **Consumption of fruit and/or vegetables** | | | | **0.0343** |  |  |
| < 5 portions | 36(17.4%) | 1 | | - |  | - |
| ≥ 5 portions | 93(11.6%) | 0.6[0.4-1.0] | | 0.0266 | 0.8[0.5-1.2] | .2141 |
| **Obesity** | | | | | | |
| **Age classes** | | |  | **<0.0001** |  |  |
| 15 - 24 | 40(8.0%) | 1 | | - | - |  |
| 24 - 34 | 111(18.4%) | 2.6[1.8-3.9] | | <0.0001 | 2.4[1.7-3.5] | **<.001** |
| ≥ 35 | 35(25.4%) | 3.9[2.4-6.4] | | <0.0001 | 4.1 [2.5-6.8] | **<.001** |
| **Area** |  |  | |  |  |  |
| Urban | 140(20.6%) | 1 | | - |  |  |
| Rural | 46(8.2%) | 0.3[0.2-0.5] | | **<0.001** | 0.3[0.2-0.5] | **<.001** |
