# Supplementary material for: High prevalence of cardiovascular risk factors in pregnant women in Benin
Source: PLoS One. 2024 Nov 14;19(11):e0310408. doi: 10.1371/journal.pone.0310408 (PMC11563467; doi:10.1371/journal.pone.0310408)
Supplement: Attachment — Submitted filename: Response to reviewers.docx [file pone.0310408.s002.docx]

**Response to reviewers**

We extend our sincere gratitude to reviewers for their corrections, contributions, and recommendations, which undoubtedly enhance the quality of our work. All their suggestions have been incorporated into the revised version of the manuscript. Therefore, we will only address the points requiring clarification in the following lines.

**Response to reviewer #1:**

1. **Can you please clarify the univariate and multivariate regression analysis and the variables that are used in the mutlivariate regression analysis? Perhaps a stepwise multivariate regression analysis would be a good approach.**

Indeed, we performed a stepwise descending multivariable logistic regression, and the details have been provided in the revised version of the manuscript. Details have been provided in the revised version of the manuscript.

2. **Can you please add discussion about limitations such as sampling bias into this study?**

A discussion on the potential sampling bias of the study has been added in the strengths and limitations section in the revised manuscript:

In Benin, according to the latest national surveys, 48% of women of childbearing age did not make at least 4 prenatal visits during pregnancy, whereas WHO recommendations stipulate a minimum of 8 visits. This could introduce a sampling bias in this study; the results may reflect the situation in the general population, however the generalization of the results to the whole country calls for caution.

3. **Can you please clarify why you had no follow-up time and why you specifically set the gestational week to be less than 20?**

We did not conduct follow-up as the study was a cross-sectional study, not a cohort study. The aim of this study was to determine the frequencies of cardiovascular risk factors before pregnancy that predispose to preeclampsia during pregnancy. According to the recommendations of scientific societies and as specified in the introduction, hypertension or diabetes detected in the first trimester (before 20 weeks of gestation) are due to preexisting chronic disorders. Similarly, in pregnant women, mid-upper arm circumference is a measure that does not vary significantly during the first weeks of pregnancy and can be used to assess overweight and obesity prior to pregnancy.

4. **Please include ethic approval numbers and dates**

The study was approved by the ethics and health sciences research committee of Parakou University (Benin) on 08th January 2020 (REF:0594/CLERB-UP/P/SP/P/SA).

We have included this in the revised version of the manuscript.

5. "**Blood pressure was measured by an electronic device"- for all women? None were measured manually, perhaps for instance in the setting of atrial fibrillation? Was the electrinic devices calibrated and checked for accuracy?**

Blood pressure was measured using an electronic device for all women. We did not perform manual measurements. All measurements were conducted following the recommendations of the STEPwise approach to NCD risk factor surveillance (STEPS). The electronic devices were calibrated, and their accuracy was verified before data collection.

Precisions were included this in the revised version of the manuscript.

6. **Conclusion: The frequency of CVRF in women before 20th week of gestation was high. Can you please rewrite and avoid "high" since it is subjective.**

“High” was remplaced by “significant” in conclusion.

**Response to reviewer #2:**

**Major comments**

1. **Page 6; line 119: What is the basis for the definitions** **of overweight and obesity in Brachial circumference?**

The definitions of overweight and obesity in Brachial circumference are based on the study published by Ahminah et al. in 2017 in the South African Medical Journal titled: "Mid-upper arm circumference: A surrogate for body mass index in pregnant women." This definition was used by authors in previous study and in the same context like us.

2. **The author should mention the difference between urban and rural backgrounds in the results of Table 1.**

The differences between rural and urban settings have been mentioned in materials and methods sections of the study on page 5, lines 97 and 98.

The title of table 1 has been corrected: Table 1: Global sociodemographic characteristics of the women according to area

**3. Page 9; line 157: Which is more accurate to say that the patient had normal blood pressure but was receiving treatment or that the patient had normal blood pressure because of the treatment?**

In stating 'the patient had normal blood pressure but was receiving treatment,' we aimed to provide information regarding the group of hypertensive patients who were on treatment before the study and had normal blood pressure at the time of the study. The sentence was modified.

**4. In Table 2, the definition of the classification of salt intake should be clarified as to how many grams of salt or sodium it is equivalent to.**

We are unable to provide information on the grams of salt in the context of our study (African countries with a significant frequency of illiterate women). Available resources did not allow for measurements to be taken. Therefore, data were collected based on the self-reports of patients and according to their perception, as detailed in the table. Advanced nutritional studies in this population indeed seem necessary.

**5. In Table 4, it is necessary to indicate which confounding factors were adjusted for.**

Confounding factors were adjusted for age and place of residence in the model. This was specified in the revised version of the article (page 6, line 168) and in Table 4.

**6. In Table 4, it is necessary to show the relationship between overweight, obesity, smoking, and alcohol consumption as factors associated with hypertension in pregnant women.**

We included theses variables in our analysis except for obesity (Supporting information), which was treated separately to avoid confounding biases. However, they did not emerge as associated with hypertension in the univariate analysis and all had p-values greater than 0.20. Therefore, we did not include them in the multivariate analysis or present them in the study, as our primary objective was to provide frequencies of cardiovascular risk factors found by other studies to be associated with preeclampsia (hypertension, diabetes, and obesity). Below are the results of analyses.

- Hypertension and tobacco consumption before pregnancy: P=0.2998
- Hypertension and tobacco consumption during pregnancy: P=1.0000
- Hypertension and alcohol consumption before pregnancy: P=0.2680
- Hypertension and alcohol consumption during pregnancy: P=0.6535

7. **Author should mention limitations and strengths in the discussion.**

Limitations and strengths were mention in revised versions (page 15, line 237).

**Minor comments:**

**1. Page 9; line 157: Twenty-eight women (2.3%) →28 (2.3%)**

The comment has been taken into account.
**2. Page 9; lines 161-163: Add (%) after the number of subjects.**

The comment has been taken into account. **3. In Table 4, the reference should be shown for each variable**

The reference categories have been specified in the tables by indicating a value of 1 for the odds ratios (OR).
